# A Small Mass Causing a Big Problem: Severe Aortic Regurgitation Induced by a Papillary Fibroelastoma

**DOI:** 10.7759/cureus.105348

**Published:** 2026-03-16

**Authors:** John Bajouka, Rana Afram, Rahul Deshmukh

**Affiliations:** 1 Internal Medicine, Henry Ford Health System, Southfield , USA; 2 Internal Medicine, Henry Ford Health System, Southfield, USA; 3 Internal Medicine, Henry Ford Providence Hospital, Southfield, USA

**Keywords:** cardiac papillary fibroelastoma, echocardiography, heart failure, mechanical malcoaptation, severe aortic regurgitation

## Abstract

Papillary fibroelastoma (PFE) is a benign primary cardiac tumor arising from the endocardial surface, most commonly affecting the valvular endocardium. Although often incidental, PFEs may lead to serious complications, including systemic embolization, valvular obstruction, or regurgitation. Aortic valve fibroelastoma resulting in severe aortic insufficiency (AI) may be rare. A 77-year-old woman with a history of hypertension and chronic kidney disease presented with progressive dyspnea, orthopnea, and bilateral lower extremity edema. Examination revealed a diastolic murmur at the left lower sternal border. Transthoracic echocardiography demonstrated a tri-leaflet aortic valve with preserved left ventricular systolic function and moderate eccentric aortic regurgitation due to incomplete cusp coaptation. A transesophageal echocardiogram was performed, which revealed a small, filamentous, and mobile mass that was visualized at the base of the noncoronary cusp (NCC), consistent with a PFE. There were no clinical or laboratory findings suggestive of infection, and the lesion’s morphology was inconsistent with vegetation. Holodiastolic flow reversal in the descending aorta confirmed severe AI. The patient was diagnosed with acute decompensated heart failure secondary to severe aortic regurgitation. After medical optimization, she underwent surgical aortic valve replacement. Intraoperative histopathology confirmed the presence of a fibroelastoma originating from the NCC. Postoperative recovery was uneventful, and the patient demonstrated marked symptomatic improvement. This case illustrates a rare presentation of PFE causing severe aortic regurgitation through mechanical interference with cusp closure rather than leaflet destruction. PFEs are typically small, avascular, and pedunculated, and when located near the coaptation zone, they can prevent complete leaflet closure, resulting in significant hemodynamic compromise. Echocardiography remains the diagnostic modality of choice, and multimodal imaging may aid in differentiating PFEs from vegetations or thrombi. Surgical excision is recommended for symptomatic, mobile, or left-sided PFEs due to the risk of embolization and valvular dysfunction. Although rare, PFE of the aortic valve should be recognized as a potential cause of severe AI. Early diagnosis and timely surgical management are curative and prevent embolic complications.

## Introduction

Primary cardiac tumors are exceedingly rare, with papillary fibroelastomas (PFEs) now recognized as the most common benign primary cardiac neoplasm in adults. PFEs most commonly affect the valvular endocardium in approximately 88% of cases [1]. PFEs are often asymptomatic and discovered incidentally during imaging or autopsy. However, their clinical significance arises from their potential to embolize or, less commonly, to physically obstruct valvular function [2]. In the aortic position, a PFE can lead to aortic insufficiency (AI), but the progression to severe, acute-on-chronic heart failure is an uncommon presentation. This case underscores the importance of recognizing how a small mass can cause "large problems" by acting as a mechanical wedge, particularly when interacting with the structural changes of an aged heart valve.

## Case presentation

A 77-year-old woman presented to the emergency department with progressive dyspnea, orthopnea, and significant bilateral lower extremity edema. Her medical history was notable for hypertension and chronic kidney disease. She was noted to have recurrent admissions for dyspnea. Upon physical examination, the patient was tachypneic with a respiratory rate of 24 breaths/minute and hypertensive with a blood pressure of 158/72 mmHg. Cardiac auscultation revealed a prominent grade 3/6 decrescendo diastolic murmur loudest at the left lower sternal border. Laboratory results showed an elevated B-type natriuretic peptide level at 3,001 pg/mL, consistent with acute heart failure, but no leukocytosis or positive blood cultures to suggest infective endocarditis (Table 1).

**Table 1:** Initial vital signs and laboratory data on presentation NT-proBNP: N-terminal prohormone of brain natriuretic peptide

Parameter	Value	Reference range
Respiratory rate	24 breaths/minute	12-20 breaths/minute
Blood pressure	158/72 mmHg	<120/80 mmHg (normal adult)
Heart rate	66 beats/minute	60-100 beats/minute (normal adult)
Oral temperature	37.1°C	36.1-37.2°C
White blood cell count	5.7 × 10³/µL	4.0-11.0 × 10³/µL
Hemoglobin	11.8 g/dL	13.5-17.5 (male)/12.0-15.5 (female) g/dL
Creatinine	1.6 mg/dL	0.6-1.3 mg/dL
Troponin (initial)	20 ng/L	<14 ng/L (assay dependent)
Troponin (repeat)	18 ng/L	<14 ng/L (assay dependent)
NT-proBNP	3,001 pg/mL	<125 (age of <75 years)/<450 (age of >75 years) pg/mL

**Figure 1:**
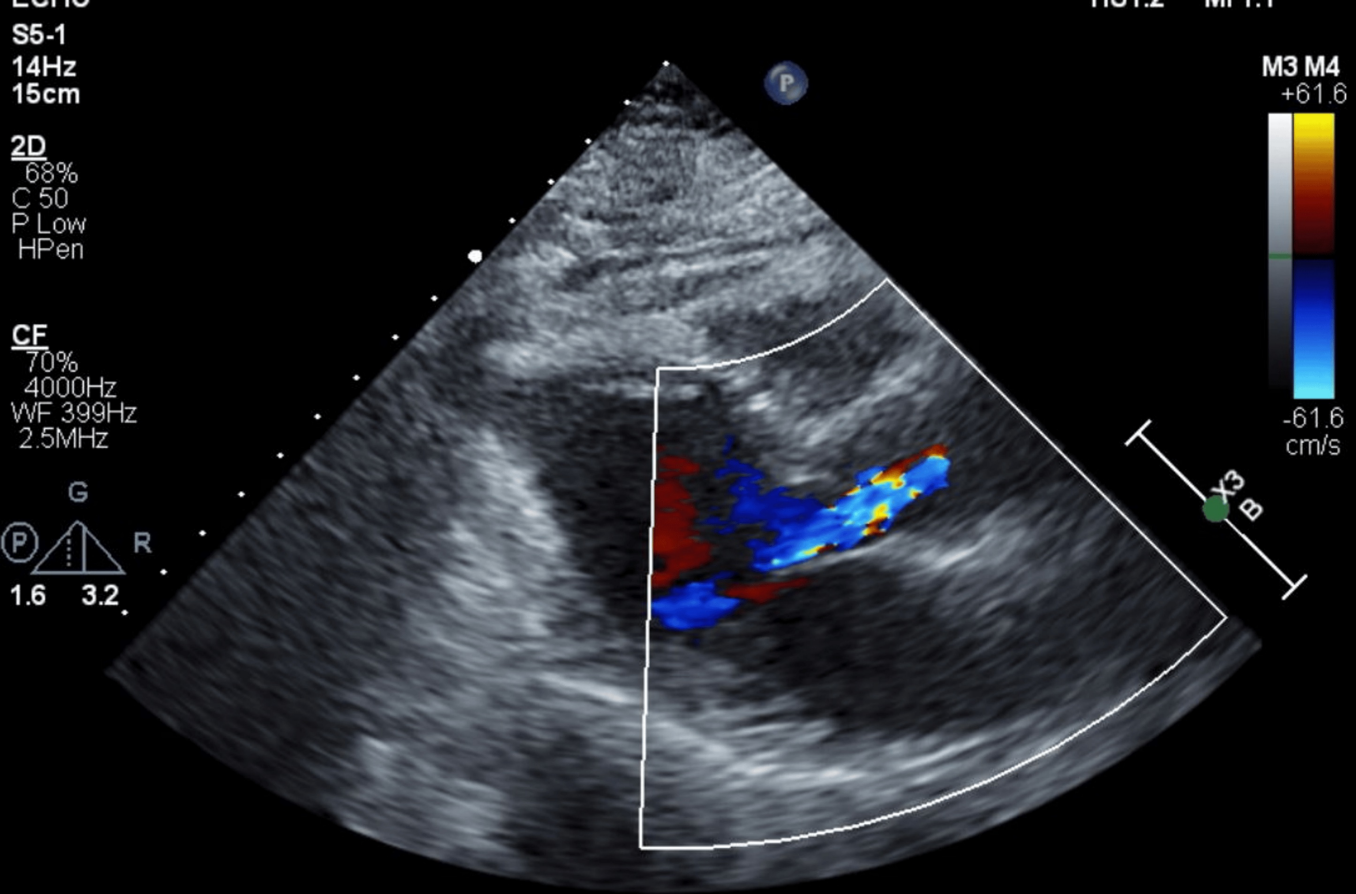
A transthoracic echocardiogram displaying a parasternal long-axis view with color Doppler showing mild-to-moderate aortic regurgitation

A transthoracic echocardiogram obtained during her prior admission approximately one month earlier showed preserved left ventricular systolic function with moderate aortic valve regurgitation (Figure 1).

**Figure 2:**
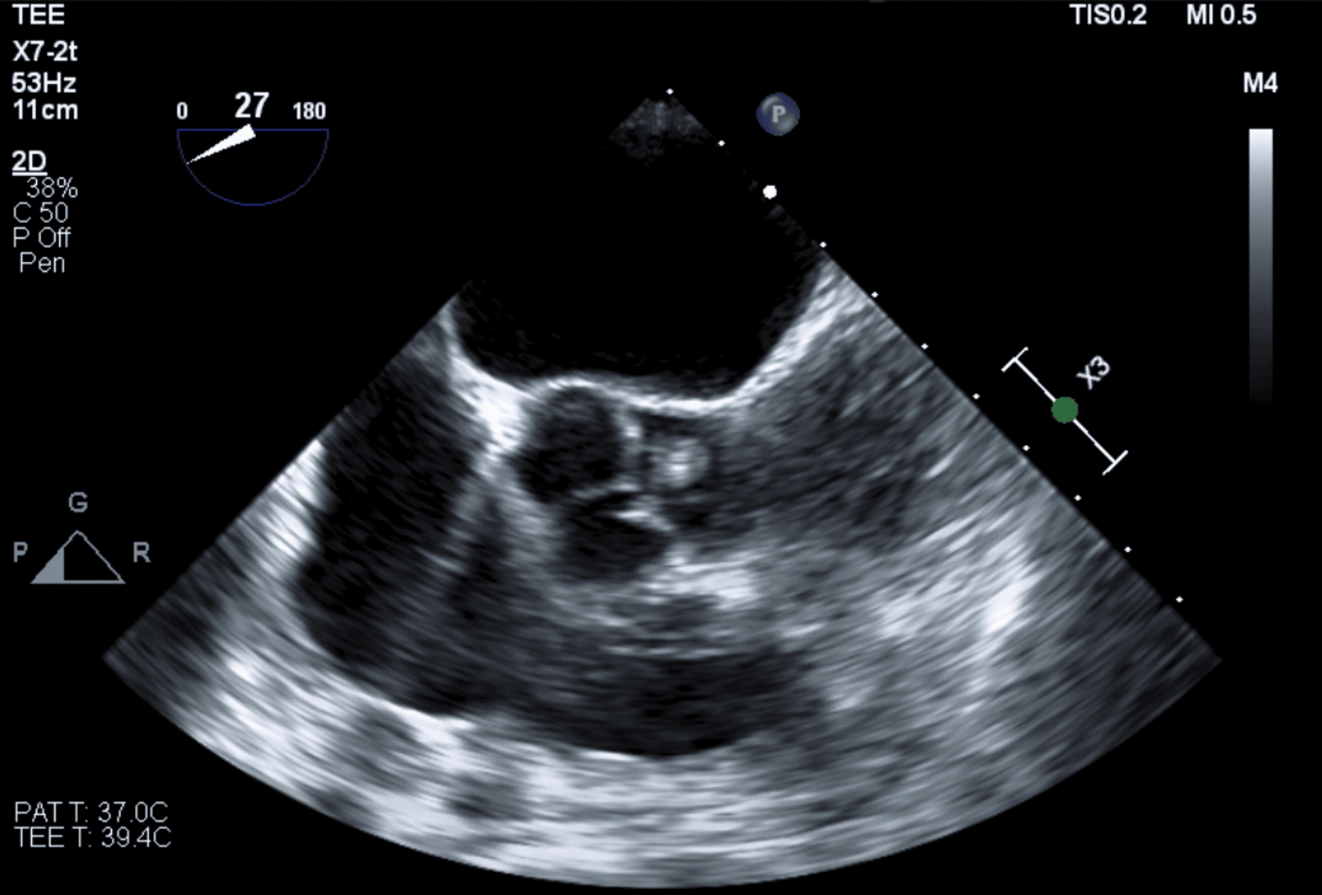
A transesophageal echocardiogram showing the mid-esophageal aortic valve short-axis view at approximately 27° A transesophageal echocardiogram shows a small, highly mobile, filamentous mass that is identified at the base of the noncoronary cusp

Given recurrent admissions for dyspnea and a previous finding of mild to moderate aortic regurgitation, a transesophageal echocardiogram was obtained. It demonstrated a tri-leaflet aortic valve with evidence of age-related degenerative thickening and mild calcification. A small, highly mobile, filamentous mass was identified at the base of the noncoronary cusp (NCC) (Figure 2).

**Figure 3:**
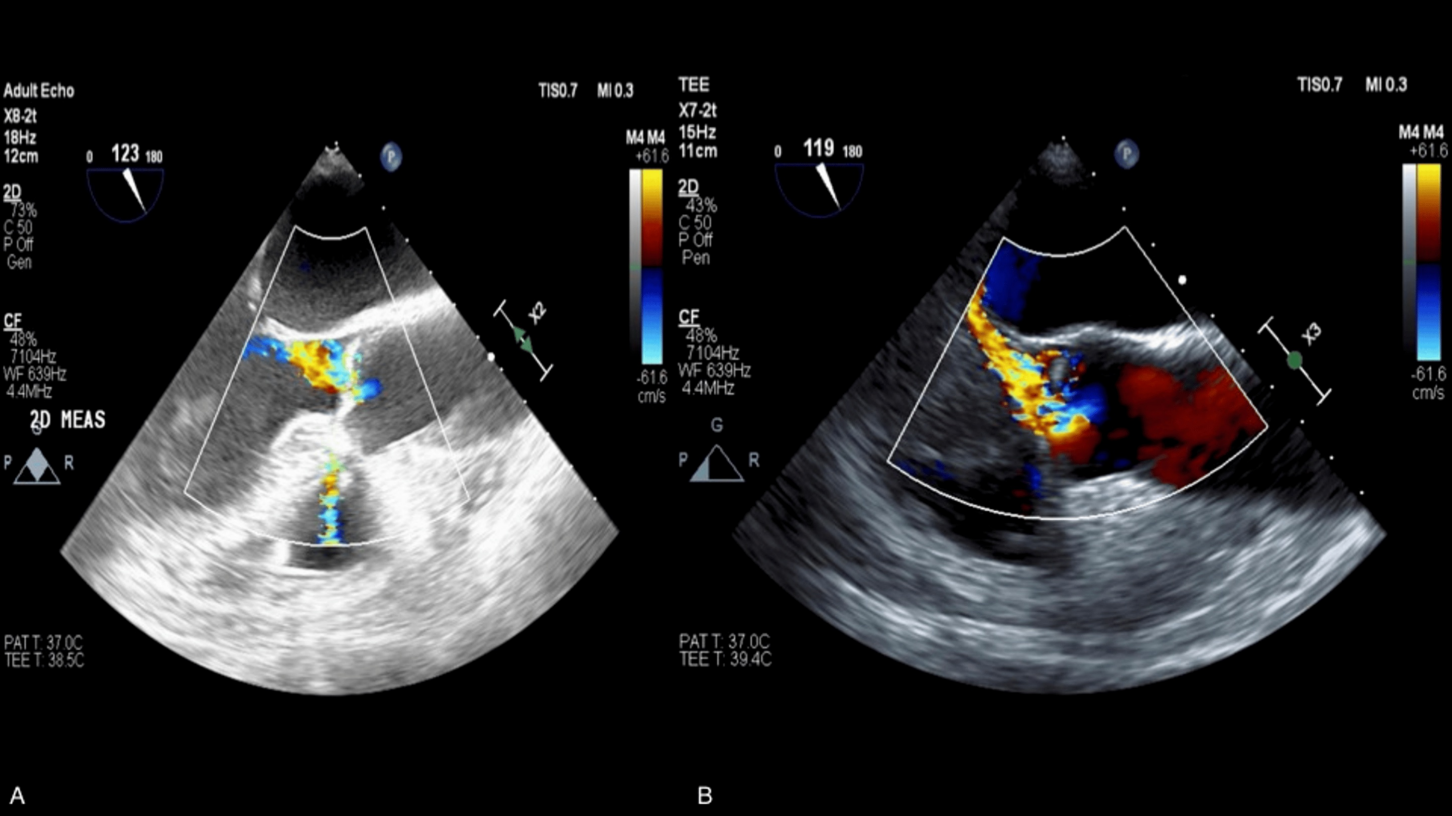
A transesophageal echocardiogram showing the mid-esophageal long-axis view at approximately 120° with color Doppler (A) Severe eccentric aortic valve regurgitation is seen at the noncoronary cusp, with leaflet bowing noted. (B) Severe eccentric aortic valve regurgitation is seen at the noncoronary cusp

During diastole, the mass was observed pressing firmly against the NCC, causing the leaflet to bow significantly into the left ventricular outflow tract (LVOT). This bowing prevented the NCC from meeting the coronary cusps at the coaptation plane. The resulting holodiastolic flow reversal confirmed severe AI (Figure 3). Interestingly, the mechanism of severe AI was not due to leaflet perforation but rather a dynamic mechanical interference.

**Figure 4:**
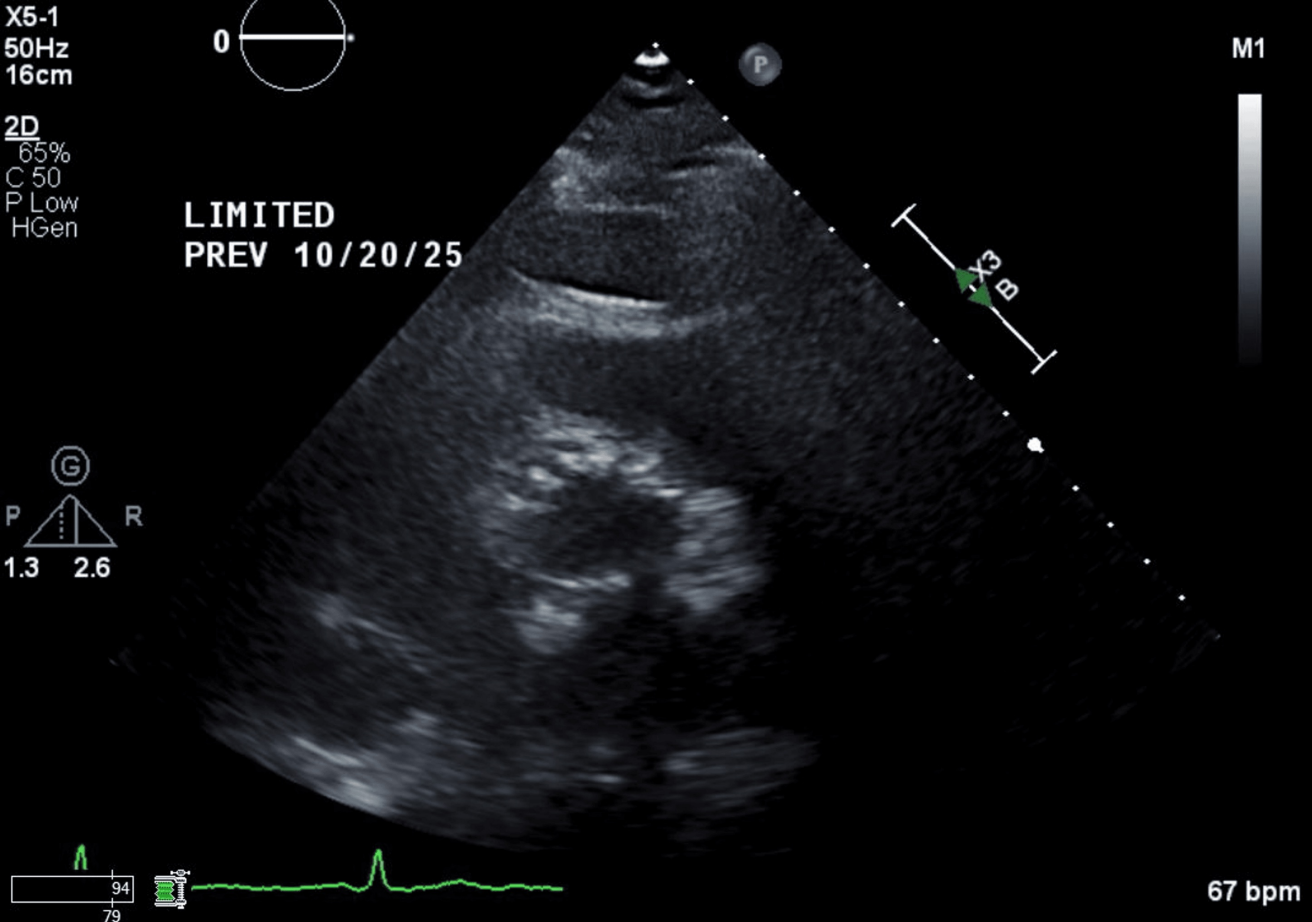
A transthoracic echocardiogram showing the parasternal short-axis view at the level of the aortic valve Post-aortic valve replacement transthoracic echocardiogram demonstrating increased echogenicity around the aortic annulus with a well-seated prosthetic valve

The patient was stabilized with intravenous diuretics for the management of severe AI. Given the severity of the symptoms and the high risk of further embolic events, she was referred for surgical intervention. Intraoperative findings confirmed a classic PFE attached to the NCC of a degenerative valve. The surgical team opted for a 27-mm bioprosthetic aortic valve replacement rather than simple excision to ensure future valvular competence (Figure 4).

The patient’s postoperative course was uneventful, and she was discharged with a marked improvement in her functional status.

## Discussion

The clinical significance of a PFE often does not correlate with its histologic size, but rather is primarily determined by its mobility and anatomical location [3]. In this case, the PFE, despite measuring less than 1 cm, precipitated a catastrophic hemodynamic decline. The tumor functions as a focal point of resistance against the valve leaflet. It is noted that, during diastole, the mass is driven into the LVOT. In a high-pressure environment, the PFE does not merely sit on the surface; it exerts a vector of force that displaces the base of the valve cusp. This localized shift may have disrupted the overall structure of the valve, ultimately leading to secondary malcoaptation. Unlike the "ball-valve" obstruction seen in larger myxomas, the PFE in this scenario induces valvular incompetence through structural redirection rather than simple physical bulk [4].

The hemodynamic severity of the resulting AI is fundamentally a product of the "aged valve" substrate, where diminished structural elasticity amplifies the mechanical impact of the mass [5]. In a younger, more pliable valve, the high elastin-to-collagen ratio allows the leaflet to deform elastically around a small mass, thereby maintaining a sufficient zone of coaptation. However, as the valve undergoes degenerative thickening and loses its inherent redundancy, its mechanical reserve may be depleted [6]. The aged NCC, characterized by decreased elasticity and structural stiffening, lacks the pliability to compensate for the presence of the tumor. Consequently, when the PFE presses against the leaflet, the entire cusp is forced to bow rather than mold. This transition from elastic deformation to structural failure may explain why even a subcentimeter mass can cause a total loss of the coaptation in older patients, whereas a similar mass might remain clinically silent in a younger individual.

Distinguishing this from other etiologies, such as infective endocarditis (IE), is critical for surgical planning and diagnostic accuracy. In IE, valvular regurgitation is typically a consequence of tissue destruction, such as perforations or erosions, or large vegetations that physically prevent closure [7]. Conversely, aortic regurgitation associated with PFEs is typically a mechanical or structural complication rather than a functional one. PFEs can cause valvular dysfunction, including aortic regurgitation, but this occurs through direct mechanical interference with valve closure rather than functional impairment [8].

This case suggests that clinicians must maintain a high index of suspicion for PFEs in elderly patients presenting with rapidly progressive heart failure, even when initial imaging identifies only small masses. The diagnosis and management of PFEs in elderly patients should prioritize tumor mobility and left-sided location over size, as these factors, not tumor dimensions, predict adverse outcomes. While rapidly progressive heart failure is not a typical presentation of PFEs [9], the approach to diagnosis and risk stratification remains consistent regardless of clinical presentation.

Given the high-stakes nature of these lesions, the clinical mandate shifts toward early surgical intervention as both a definitive treatment for mechanical heart failure and a critical prophylaxis against systemic embolization. Unlike other cardiac tumors where a waiting approach might be considered for small, asymptomatic masses, the PFE carries a disproportionate risk of stroke or myocardial infarction due to its fragile, thrombus-prone surface and high mobility [10]. Ultimately, prompt recognition and surgical excision can convert a potentially devastating clinical course into a definitive cure, underscoring the principle that when a mobile left-sided intracardiac mass is identified, its location and mobility should take precedence over size alone in guiding the urgency of management [11].

## Conclusions

PFE of the aortic valve is a critical, although rare, differential diagnosis in patients presenting with unexplained severe AI. As demonstrated, the interaction between a mobile mass and an aged, degenerative valve can lead to mechanical malcoaptation through diastolic bowing. Early surgical intervention is not only curative for AI but also serves as a necessary preventative measure against systemic embolization. Timely diagnosis via multimodal imaging remains the gold standard for managing these small masses with potentially life-threatening consequences.

## References

[1] Maleszewski JJ, Bois MC, Bois JP, Young PM, Stulak JM, Klarich KW (2018). Neoplasia and the heart: pathological review of effects with clinical and radiological correlation. J Am Coll Cardiol.

[2] Anastacio MM, Moon MR, Damiano RJ Jr, Pasque MK, Maniar HS, Lawton JS (2012). Surgical experience with cardiac papillary fibroelastoma over a 15-year period. Ann Thorac Surg.

[3] Gowda RM, Khan IA, Nair CK, Mehta NJ, Vasavada BC, Sacchi TJ (2003). Cardiac papillary fibroelastoma: a comprehensive analysis of 725 cases. Am Heart J.

[4] Zull DN, Diamond M, Beringer D (1985). Angina and sudden death resulting from papillary fibroelastoma of the aortic valve. Ann Emerg Med.

[5] Grande KJ, Cochran RP, Reinhall PG, Kunzelman KS (1999). Mechanisms of aortic valve incompetence in aging: a finite element model. J Heart Valve Dis.

[6] Raisi-Estabragh Z, Szabo L, Schuermans A (2024). Noninvasive techniques for tracking biological aging of the cardiovascular system: JACC family series. JACC Cardiovasc Imaging.

[7] Pawar S, Allen C, Mori M (2026). Contemporary diagnosis and treatment of aortic regurgitation: a state-of-the-art review. J Am Coll Cardiol.

[8] Carino D, Nicolini F, Molardi A, Indira Dadamo C, Gherli T (2017). Unusual locations for cardiac papillary fibroelastomas. J Heart Valve Dis.

[9] Schlingmann TR, Gauvreau K, Colan SD, Powell AJ (2015). Correction of Doppler gradients for pressure recovery improves agreement with subsequent catheterization gradients in congenital aortic stenosis. J Am Soc Echocardiogr.

[10] Tamin SS, Maleszewski JJ, Scott CG (2015). Prognostic and bioepidemiologic implications of papillary fibroelastomas. J Am Coll Cardiol.

[11] Butany J, Nair V, Naseemuddin A (2005). Cardiac tumours: diagnosis and management. Lancet Oncol.

